# Analysis of 358 histopathological reports of oral and maxillofacial lesions in elderly patients from Tanzania: a cross-sectional study

**DOI:** 10.1097/MS9.0000000000000597

**Published:** 2023-04-07

**Authors:** Karpal S. Sohal, Boniphace M. Kalyanyama, Ashu M. Agbor

**Affiliations:** a Department of Oral and Maxillofacial Surgery, Muhimbili University of Health and Allied Sciences, Dar es Salaam, Tanzania; b School of Dentistry, University of Montagnes, Bangangte, Cameroon

**Keywords:** geriatric dentistry, geriatrics, incidence, oral and maxillofacial pathology, Tanzania

## Abstract

In Tanzania, roughly 6% of the total population is elderly, which is the age group that is vulnerable to several diseases in the orofacial region. This study aimed to determine the incidence of oral and maxillofacial lesions in elderly Tanzanian patients.

**Material and Methods:**

This was a cross-sectional study of histopathological results of patients with oral and maxillofacial lesions attended at Muhimbili National Hospital. All patients aged 60 years and above diagnosed with oral and maxillofacial lesions between 2016 and 2021 were included in the study. The information gathered included the age and sex of the patients, histopathological diagnosis, and anatomical location of the lesion. The Statistical Package for the Social Sciences, version 26 computer program was used for data analysis.

**Results:**

A total of 348 histopathological reports of 348 elderly patients with oral and maxillofacial lesions were obtained. There was an equal distribution by sex. Majority (78.2%) of the lesions were malignant, followed by benign ones (12.6%). The frequently affected site was the tongue (18.1%) and the mandible (15.4%). Squamous cell carcinoma was the most (60.3%) frequently encountered lesion. Others included adenoid cystic carcinoma (5.5%) and ameloblastoma (3.7%).

**Conclusions:**

The burden of oral and maxillofacial lesions among the elderly Tanzanian population was substantial. There was no sex predilection. A majority of the lesions were malignant, and the tongue was the frequently involved site.

## Introduction

Highlights

- The burden of oral and maxillofacial lesions among the elderly Tanzanian population is substantial.
- Elderly males and females are equally affected by oral and maxillofacial lesions in Tanzania.
- A majority of the oral and maxillofacial lesions among the elderly Tanzanian population are malignant, and the tongue is the frequently involved site.

With aging, an individual becomes vulnerable to several diseases including those involving the orofacial region^1^,^2^. This vulnerability to orofacial lesions has led to a rise in interest in the oral health of this population and hence an increase in epidemiological surveys in this age group^2^,^3^.

Globally, the proportion of elderly individuals is increasing^2^. In Tanzania, roughly 6% of the total population are individuals aged 60 years and above, and it is projected this percentage will increase by up to two-fold by 2050^4^. Reports from sub-Saharan Africa (including Tanzania) show that the pattern of disease among elderly patients is shifting from infectious diseases to noncommunicable diseases (orofacial lesions being inclusive)^5^–^7^.

Though many studies throughout the world report on oral pathologies in the elderly population, most are based on clinical presentation rather than histological diagnosis^8^–^10^. The pattern of oral and maxillofacial lesions differs between countries^11^. Mohan *et al*.^9^ reported majority of biopsied lesions in the Indian population were malignant and premalignant lesions. While in Brazil^2^, inflammatory/reactive lesions followed by malignant lesions were frequent histological diagnoses, a report from Iran shows non-neoplastic conditions were most prevalent^12^. Akinshipo *et al*.^13^ found that malignant and non-neoplastic lesions were prevalent histological diagnoses among the elderly in Nigeria.

The variations in the occurrence of oral and maxillofacial lesions from one country to another call for locally tailored treatment needs for a particular nation. Therefore, it is practical to conduct epidemiologic studies to provide information that is important to understand the pattern of oral and maxillofacial disease in a specific population^14^. To the best of our knowledge, there are limited previous studies from Tanzania that focus on the pattern of histological diagnoses among the elderly population. Hence, this study aimed to determine the incidence of oral and maxillofacial lesions in the elderly population in Tanzanian.

## Methods

In this cross-sectional study, we retrospectively analyzed histopathological reports of biopsied orofacial lesions archived in the Department of Oral and Maxillofacial Surgery (OMFS) of the Muhimbili University of Health and Allied Sciences (MUHAS), Dar es Salaam, Tanzania. The inclusion criteria were histological reports of all patients aged 60 years and above attended between 2 January 2016 and 31 December 2021. Excluded criteria were any reports with inconclusive diagnoses or without a final diagnosis, and the reports of patients who were diagnosed based on the fine needle aspiration cytology.

A data collection form was used to extract information from the histopathological reports. The information captured included the age and sex of the patient, the anatomical location of the lesion, histopathological diagnosis, and histopathological identification number. In a case where a single patient had more than one result, as one for presurgery incisional biopsy and another for postsurgical excisional biopsy, then the post-operative result was included.

The data obtained from this study were coded and analyzed using Statistical Package for Social Sciences software (SPSS) for Windows (IBM Corp.). The continuous variables were presented in the form of the mean and percentages, while the categorical variables were presented in the form of tables. The participants were grouped into two age groups: younger-old (60–74 years) and older-old (75+ years). The lesions were categorized per WHO classification^15^.

To assess the relationship between sociodemographic characteristics and the nature of the lesion either one-way analysis of variance or chi-square tests were used. The α value less than 0.05 was selected for statistical significance.

Ethical clearance was sought from the MUHAS research and ethics committee (DA.25/111/01B/208), and permission to conduct the study was obtained from the appropriate authorities of the department of OMFS-MUHAS. The study fulfills the STROCSS criteria for cross-sectional studies^16^.

## Results

### Age and sex of the patients in relation to the nature of the lesion

A total of 1824 reports of patients with oral and maxillofacial lesions were retrieved. Out of these, 348 (19.1%) reports were of elderly patients. The age of patients at diagnosis ranged from 60 to 106 years, with a mean age of 70.23 (SEM=0.46) years. Most (N=248, 71.3%) were younger olds. There was an equal distribution of the patient by sex male-to-female ratio was 1:1. Majority (N=272, 78.2%) of the lesions were malignant. There was no statistically significant association between the nature of the lesions and age groups and the sex of the patients (Table 1).

**Table 1:** Distribution of elderly patients with oral and maxillofacial lesions according to age groups, sex, and the nature of the lesions

	Nature of the lesion [n (%)]				
Age and sex of the participants	Benign (N=44)	Malignant (N=272)	Cyst (N=7)	Inflammatory (N=25)	*P*
Age groups (years)					
60–74	33 (13.3)	193 (77.8)	5 (2.0)	17 (6.9)	0.931
≥75	11 (11.0)	79 (79.0)	2 (2.0)	8 (8.0)	
Age [mean (SEM)]	69.18 (1.27)	70.43 (0.52)	69.86 (3.67)	69.96 (1.90)	0.130
Sex					
Female	23 (13.2)	130 (74.7)	3 (1.7)	18 (10.3)	
Male	21 (12.1)	142 (81.6)	4 (2.3)	7 (4.0)	0.133

### Anatomical locations of oral and maxillofacial lesions in the elderly population

The location of the lesion was reported in the majority (N=337, 96.8%) of the histological reports. A total of 15 anatomical sites were affected whereby the tongue was most (N=61, 18.1%) affected followed by the mandible (N=52, 15.4%), and the buccal mucosa (N=40, 11.9%). The frequency of other sites is shown in Table 2.

**Table 2:** Distribution of study participants according to the anatomical location of the lesion and the histopathological nature of the disease

	Nature of the lesion [n (%)]				
Anatomical location of the lesion	Benign (N=44, 100%)	Malignant (N=261, 100%)	Cyst (N=7, 100%)	Inflammatory (N=25, 100%)	Total
Buccal mucosa	–	39 (14.9)	–	1 (4.0)	40
Cheek (extraoral)	2 (4.5)	4 (1.5)	–	2 (8.0)	8
Floor of the mouth	–	10 (3.8)	1 (14.3)	1 (4.0)	12
Gingiva	1 (2.3)	32 (12.3)	–	1 (4.0)	34
Lip	–	26 (10.0)	–	4 (16.0)	30
Mandible	19 (43.2)	31 (11.9)	–	2 (8.0)	52
Maxilla	9 (20.5)	8 (3.1)	2 (28.6)	–	19
Maxillary sinus	–	4 (1.5)	–	–	4
Neck	–	1 (0.4)	–	2 (8.0)	3
Orbital	–	1 (0.4)	–	–	1
Palate	3 (6.8)	30 (11.5)	2 (28.6)	1 (4.0)	36
Parotid	6 (13.6)	7 (2.7)	2 (28.6)	2 (8.0)	17
Retromolar	–	2 (0.8)	–	–	2
Submandibular	2 (4.5)	9 (3.4)	–	7 (28.0)	18
Tongue	2 (4.5)	57 (21.8)	–	2 (8.0)	61

Eleven cases of malignant lesions had missing information about the anatomical site.

Malignant lesions affected every anatomical site, whereas the palate and parotid were the only locations to be affected by all 4 types of lesions. The site frequently affected by malignant lesions were the tongue, buccal mucosa, and gingiva. The benign lesions were common in the mandible, maxilla, and parotid. The cysts were found in the maxilla, palate, and parotid, and the submandibular region and lip were the most common site for inflammatory lesions (Table 2).

### The overall histopathological diagnosis of oral and maxillofacial lesions in the elderly population

A total of 43 histopathological diagnoses were made out of the 348 tissue samples from elderly patients who had lesions involving the oral and maxillofacial region. Squamous cell carcinoma was the most (N=210, 60.3%) frequently diagnosed lesion. Other diagnoses with higher frequency were adenoid cystic carcinoma (N=19, 5.5%), ameloblastoma (N=13, 3.7%), and adenocarcinoma (N=12, 3.4%).

### The benign oral and maxillofacial lesions in the elderly population

Among the benign lesions in the elderly, 11 different histopathological diagnoses were made. The nonodontogenic lesions were the commonest (N=23, 52.3%) of which pleomorphic adenomas were predominant. Ameloblastoma, which was the only odontogenic benign tumor, accounted for more than a quarter of the benign lesions (N=13, 29.5%) (Table 3).

**Table 3:** Distribution of elderly patients with oral and maxillofacial lesions according to age groups, sex, and histopathological type of benign lesions

	Age groups [n (%)] (years)		Sex [n (%)]		
The histological type of benign lesion (N=44)	60–74	75+	Female	Male	Total [n (%)]
Odontogenic tumor (N=13, 29.5%)					
Ameloblastoma	8 (61.5)	5 (38.5)	2 (15.4)	11 (84.6)	13 (100.0)
Fibro-osseous lesions (N=8, 18.2%)					
Fibrous dysplasia	2 (100.0)	–	2 (100.0)	–	2 (100.0)
Ossifying fibroma	4 (66.7)	2 (33.3)	4 (66.7)	2 (33.3)	6 (100.0)
Nonodontogenic tumors (N=23, 52.3%)					
Pleomorphic adenoma	10 (100.0)	–	7 (70.0)	3 (30.0)	10 (100.0)
Fibroma	2 (66.7)	1 (33.3)	3 (100.0)	–	3 (100.0)
Central giant cell tumor	2 (100.0)	–	1 (50.0)	1 (50.0)	2 (100.0)
Hemangioma	2 (100.0)	–	1 (50.0)	1 (50.0)	2 (100.0)
Lipoma	2 (100.0)	–	1 (50.0)	1 (50.0)	2 (100.0)
Pyogenic granuloma	1 (50.0)	1 (50.0)	–	2 (100.0)	2 (100.0)
Chondroma	–	1 (100.0)	–	1 (100.0)	1 (100.0)
Osteoma	1 (100.0)	–	1 (100.0)	–	1 (100.0)

### The malignant oral and maxillofacial lesions in the elderly population

A total of 16 different histological diagnoses were made in 272 patients with malignant tumors. The predominant type of malignant lesion was carcinomas (N=255, 93.7%). The frequently diagnosed malignant lesion in elderly patients was squamous cell carcinoma (N=210, 77.2%) (Table 4).

**Table 4:** Distribution of elderly patients with oral and maxillofacial lesions according to age groups, sex, and histopathological type of malignant lesions

	Age groups [n (%)] (years)		Sex [n (%)]		
The histological type of malignant lesion (N=272)	60–74	75+	Female	Male	Total [n (%)]
Carcinoma (N=255, 93.7%)					
Adenocarcinoma	9 (75.0)	3 (25.0)	6 (50.0)	6 (50.0)	12 (100.0)
Adenoid cystic carcinoma	12 (63.2)	7 (36.8)	7 (36.8)	12 (63.2)	19 (100.0)
Basal cell carcinoma	1 (50.0)	1 (50.0)	1 (50.0)	1 (50.0)	2 (100.0)
Metastatic carcinoma	2 (66.7)	1 (33.3)	3 (100.0)	–	3 (100.0)
Mucoepidermoid carcinoma	2 (40.0)	3 (60.0)	2 (40.0)	3 (60.0)	5 (100.0)
Squamous cell carcinoma	155 (73.8)	55 (26.2)	102 (48.6)	108 (51.4)	210 (100.0)
Secretory carcinoma	1 (100.0)	–	1 (100.0)	–	1 (100.0)
Verrucous carcinoma	1 (33.3)	2 (66.7)	1 (33.3)	2 (66.7)	3 (100.0)
Lymphoma (N=7, 2.6%)					
Hodgkin’s lymphoma	2 (100.0)	–	–	2 (100.0)	2 (100.0)
Non-Hodgkin’s lymphoma	3 (60.0)	2 (40.0)	1 (20.0)	4 (80.0)	5 (100.0)
Sarcoma (N=3, 1.1%)					
Fibrosarcoma	–	1 (100.0)	1 (100.0)	–	1 (100.0)
Leiomyosarcoma	1 (100.0)	–	–	1 (100.0)	1 (100.0)
Osteosarcoma	1 (100.0)	–	1 (100.0)	–	1 (100.0)
Other (N=7, 2.6%)					
Kaposi’s sarcoma	–	1 (100.0)	–	1 (100.0)	1 (100.0)
Malignant melanoma	1 (33.3)	2 (66.7)	2 (66.7)	1 (33.3)	3 (100.0)
Plasmacytoma	2 (66.7)	1 (33.3)	2 (66.7)	1 (33.3)	3 (100.0)

### Inflammatory oral and maxillofacial conditions in the elderly population

A total of 25 cases had 9 different histological diagnoses of inflammatory conditions, of which the most common (N=9, 36%) was chronic inflammation (Table 5).

**Table 5:** Distribution of elderly patients with oral and maxillofacial lesions according to age groups, sex, and histopathological type of inflammatory condition

	Age groups [n (%)] (years)		Sex [n (%)]		
The histological type of inflammatory condition (N=25)	60–74	75+	Female	Male	Total [n (%)]
Acute inflammation	–	1 (100.0)	1 (100.0)	–	1 (100.0)
Chelitis granulomatosa	1 (100.0)	–	1 (100.0)	–	1 (100.0)
Chronic inflammation	7 (77.8)	2 (22.2)	7 (77.8)	2 (22.2)	9 (100.0)
Chronic sialoadenitis	3 (75.0)	1 (25.0)	1 (25.0)	3 (75.0)	4 (100.0)
Chronic ulcer	1 (50.0)	1 (50.0)	1 (50.0)	1 (50.0)	2 (100.0)
Foreign body granuloma	1 (100.0)	–	1 (100.0)	–	1 (100.0)
Lymphadenitis	–	1 (100.0)	1 (100.0)	–	1 (100.0)
Pseudoepitheliomatous hyperplasia	–	2 (100.0)	2 (100.0)	–	2 (100.0)
Suppurative inflammation	3 (100.0)	–	2 (66.7)	1 (33.3)	3 (100.0)

### The oral and maxillofacial cysts in the elderly population

A total of seven cases were diagnosed with seven different types of cystic lesions. All the cystic lesions had a single frequency of occurrence. They included a parotid cyst, dentigerous cyst, dermoid cyst, lymphoepithelial cyst, mid-palatine cyst, nasopalatine cyst, and a radicular cyst.

## Discussion

The aging population is becoming a public health concern, especially in developing countries^17^. The elderly are susceptible to noncommunicable diseases, including those involving the orofacial region^1^,^2^,^17^. In this group, the vulnerability to orofacial diseases may be attributed to aging-related changes in the tissues and the immune response^18^. Some of the changes include thinning of the oral epithelium, loss of fat, decrease in the rate of cell proliferation, and collagen fibers degeneration, which subsequently weaken the immune response to stimuli^1^. Consequently, this population is susceptible to various oral and maxillofacial lesions ranging from neoplastic to inflammatory^2^,^8^–^13^.

In this study, 19.1% of the histological reports of oral and maxillofacial lesions were of elderly patients. Our findings were almost similar to those from Brazil^1^, Taiwan^19^, and Iran^12^ but different from reports from Nigeria^13^ and India^9^. This disparity may be due to the methodological differences between various studies. The high number of elderly patients being diagnosed with oral and maxillofacial lesions in Tanzania is attributed to an increase in their number in the general population^5^.

There was an equal distribution of patients by sex in the present study. On the contrary, studies from India^9^ and Taiwan^19^ reported slightly higher male preponderance whereas, in Brazil^1^,^2^, Iran^12^, and Nigeria^13^ there were more females. The findings from the current study reflect improved sex equity in access to healthcare in Tanzania^5^. Moreover, it might be speculated that in Tanzanian society, despite the risk factors for oral and maxillofacial lesions may be similar for both sexes, biological and genetic makeup may have a role as well, and this is further augmented by the findings that the association between the nature of the lesion and the sex of the patients was statistically insignificant.

The findings of this study depicted a total of 43 different histopathological types of lesions (ranging from inflammatory conditions to malignancies) were diagnosed among the elderly. The majority of these lesions were categorized as malignant neoplasms. The higher incidence of malignancies in the elderly could be explained by the multistage modal of carcinogenesis. The modal theorizes that somatic mutation in individual cells is due to a series of genetic changes occurring due to the accumulation of carcinogens over years^20^. Whereas the occurrence of inflammatory conditions may be attributed to hormonal and metabolic changes that occur with aging, contributing to a decline in physiological functions and the development of chronic diseases^21^. Benign lesions tend to affect young individuals more than elderly^13^, possibly due to genetic alterations that occur early in life^22^. The indolent nature of most benign lesions can lead to late diagnosis since, in the early stages, majority of these lesions are asymptomatic thus patients seek no care. The patients eventually present to the health facility only when these lesions become symptomatic, hence at the time of diagnosis the patients have advanced age^23^.

In the current study, the most commonly affected anatomical location was the intraoral region which included the tongue, gingiva, buccal mucosa, palate, and floor of the mouth. This was not a surprise as the results of this study point out that the majority of the lesions were malignant, specifically carcinomas. Carcinomas were predominant because they arise from epithelial cells which line up the surface of the orofacial region, and these cells are exposed to a multitude of carcinogens that pass in large quantities through the oral route. The mandible was the second most affected site. It is the common site for several benign lesions, malignant lesions like osteosarcoma^24^,^25^, and even metastasis to the jaws^26^.

Concurring with results from previously published studies^12^,^13^,^19^,^27^ the most frequent histological diagnosis was squamous cell carcinoma. The high prevalence of squamous cell carcinoma may be ascribed to the fact that these lesions arise from the epithelial cells which are among the most abundant cells in the body found on the skin, oral mucosa, and even the salivary glands^28^. Ameloblastoma, an odontogenic tumor derived from either epithelial or mesenchymal elements or both was the second most common lesion. In general, ameloblastoma can present at any age, though most are seen within the third and fifth decades of life^11^,^29^.

In conducting the present study, some limitations were unavoidable. First, some of the lesions were not represented since most clinicians tended to rely on clinical and radiological findings for their management hence they were not sent for histopathological analysis, for example, leukoplakia, radicular cysts, periodontal cysts, etc. Second, being a retrospective study, some details like previous treatment history, associated comorbidities, and the duration of the lesion could not be obtained. Another limitation is that this study was carried out in a single centre (though it is a national referral hospital), hence generalization of results is done with caution. Nevertheless, the current study provides valuable information on oral and maxillofacial lesions in the elderly. This will enable early diagnosis and management of oral and maxillofacial lesions, subsequently helping improve the life quality of life of elderlies^1^. Moreover, the results of this study form an important stepping stone for more specific and multicentric studies involving elderly patients. The results of this study also serve as a reminder to all dental professionals not to rely solely on clinical and radiological diagnoses of orofacial lesions, but it is worth getting every lesion excised (no matter how small) for histopathological analysis for confirming the diagnosis.

## Conclusions

The burden of oral and maxillofacial lesions among the elderly in the Tanzanian population according to this study was substantial. This group of the population was prone to an array of lesions with the majority being malignant. Generally, the tongue, mandible, and buccal mucosa were the frequently affected sites. While squamous cell carcinoma was the most common malignant condition affecting the elderly population, ameloblastoma, and pleomorphic adenomas were frequently biopsied benign lesions.

## Ethical approval

The approval for this study was obtained from the Institutional Review Board (Ref no: DA.25/111/01B/208).

## Consent

NA.

## Sources of funding

None.

## Author contribution

K.S.S.: conceived the idea, guided the whole process of data collection, data analysis, writing and editing of the first draft, and approved the final draft. B.M.K.: broadened the conceived idea, guided the whole process of data collection and writing and editing of the first draft, and approved the final draft. A.M.A.: was involved in data analysis, writing the first draft and its improvement, and approved the final draft.

## Conflicts of interest disclosure

The authors declare that they have no financial conflict of interest with regard to the content of this report.

## Research registration unique identifying number (UIN)

DA.25/111/01B/208.

## Guarantor

Karpal S. Sohal.

## Provenance and peer review

Not commissioned, externally peer-reviewed.
